# Discovery and Characterization of a Novel Carlavirus Infecting Potatoes in China

**DOI:** 10.1371/journal.pone.0069255

**Published:** 2013-06-21

**Authors:** Yuan-Yuan Li, Ru-Nan Zhang, Hai-Ying Xiang, Hesham Abouelnasr, Da-Wei Li, Jia-Lin Yu, Jenifer Huang McBeath, Cheng-Gui Han

**Affiliations:** 1 State Key Laboratory for Agro-biotechnology and Department of Plant Pathology, China Agricultural University, Beijing, P. R. China; 2 Plant Pathology and Biotechnology Laboratory, Agriculture and Forestry Experiment Station, University of Alaska Fairbanks, Fairbanks, Alaska, United States of America; Jagiellonian University, Poland

## Abstract

A new carlavirus, tentatively named Potato virus H (PVH), was found on potato plants with mild symptoms in Hohhot, Inner Mongolia Autonomous Region, China. PVH was confirmed by genome sequencing, serological reactions, electron microscopy, and host index assays. The PVH particles were filamentous and slightly curved, with a modal length of 570 nm. Complete RNA genomic sequences of two isolates of PVH were determined using reverse transcription-PCR (RT-PCR) and the 5′ rapid amplification of cDNA ends (5' RACE) method. Sequence analysis revealed that PVH had the typical genomic organization of members of the genus *Carlavirus*, with a positive-sense single-stranded genome of 8410 nt. It shared coat protein (CP) and replicase amino acid sequence identities of 17.9–56.7% with those of reported carlaviruses. Phylogenetic analyses based on the protein-coding sequences of replicase and CP showed that PVH formed a distinct branch, which was related only distantly to other carlaviruses. Western blotting assays showed that PVH was not related serologically to other potato carlaviruses (*Potato virus S*, *Potato virus M*, and *Potato latent virus*). PVH systemically infected *Nicotiana* *glutinosa* but not *Nicotiana tabacum*, *Nicotiana* *benthamiana* , or *Chenopodium* *quinoa* , which is in contrast with the other potato carlaviruses. These results support the classification of PVH as a novel species in the genus *Carlavirus*. Preliminary results also indicated that a cysteine-rich protein encoded by the smallest ORF located in the 3' proximal region of the genome suppressed local RNA silencing and enhanced the pathogenicity of the recombinant PVX.

## Introduction

Potato is one of the most important food crops in the world. Potato production reached a record level of 374.38 million tons in 2011 and worldwide potato production has exceeded the growth in production of other major food commodities in developing countries, particularly in Asia. With a record production of 88.35 million tons in 2011, China was the world’s largest potato-producing country (FAO, 2011).

Potato production is affected by many virus diseases. In addition to the well-studied potato viruses such as Potato virus X (PVX), Potato virus Y (PVY), and Potato leaf roll virus (PLRV), which cause severe symptoms and yield losses, Potato virus S (PVS) and Potato virus M (PVM) are common but produce mild symptoms, although they also adversely affect yield. Losses of 10–15% have been reported in individual plants from susceptible cultivars with PVS secondary infection, and the infected plants may yield a higher proportion of unmarketable small tubers. The yield losses of PVM-secondarily infected plants may be around 10% or higher [1]. PVM and PVS both belong to the genus *Carlavirus* in the newly established family *Betaflexiviridae* [2].

The genus *Carlavirus* comprises 43 species and PVS [3], PVM [4], *Potato latent virus* (PotLV) [5], and Potato virus P (PVP) (also known as *Potato rough dwarf virus*, PRDV) [6,7] have been reported to infect potatoes [8,9]. The genome organization of carlaviruses typically comprises six open reading frames (ORFs) with short untranslated regions (UTRs) located at the 5' and 3' termini of the genome. ORF1 encodes a polypeptide that functions as the viral replicase; ORFs 2, 3, and 4 form the triple gene block (TGB) and encode polypeptides that facilitate virus movement; ORF5 encodes the coat protein (CP) and overlaps with ORF6, which encodes a cysteine-rich protein (CRP) [2]. Recent studies of PVS and PVM have shown that the CRP and the characteristic TGB might both play important roles in the infection process of carlaviruses and their interactions with other viruses [10-13]. All known carlavirus species can be transmitted mechanically [2], while PVS [14-16], PVM [17], and PoLV [5] have also been reported to be transmissible in a non-persistent manner by *Myzus* *persicae* .

In China, viral diseases are a major limiting factor of potato production. Eleven viral species (approximately 40 have been reported worldwide [18,19]) have been found to infect potato and six of these viruses are recognized as major viruses: PLRV, Potato virus A (PVA), PVM, PVS, PVX, and PVY [20]. PVS is the most prevalent and is found in the majority of potato producing regions, including Guangdong (southern winter crop zone), Hebei (central double crop zone), Inner Mongolia, Heilongjiang, Jilin (northern single crop zone), Yunnan, Guizhou, Chongqing, and Sichuan (southwestern mixed crop zone) [21-25]. PVS is usually found in mixed infestation with PVX, PVM, PLRV, and PVY [24,25]. Yield losses of 10-20% due to PVS have been reported [24,26] while mixed infections involving PVM cause 20-30% losses of yield [24]. There are fewer reports of PVM in China [23,27]. There have been no reports of PoLV or PVP in China.

Our studies of potato viruses in Inner Mongolia led to the discovery of a new virus. The determination and analysis of its partial nucleotide sequence provided evidence that this agent may represent a new species of the genus *Carlavirus*. To characterize this novel carlavirus at the molecular, serological, and biological levels, we determined its complete genomic sequence, serological reaction assay results, and host range.

## Materials and Methods

### Virus source and plant material

From August 2009 to November 2012, 171 potato field samples were collected from six provinces in China, including Hebei in the central double crop zone; Inner Mongolia, Xinjiang, Heilongjiang, and Liaoning in the northern single crop zone; and Yunnan in the southwestern mixed crop zone. Potato plants infected with PVH from Qujing were used for mechanical inoculations. Inocula were verified by RT-PCR and western blotting to confirm purity. Virus particles for electron microscopy were prepared from PVH-infected *Nicotiana* *glutinosa* . The potato cv. Shepody plants and *Solanum* *lycopersicum* tissue culture seedlings used in the bioassays were kindly provided by Professor Liyun Guo and Heng Jian of the China Agricultural University (CAU), respectively. Other plant materials ( *Chenopodium* *amaranticolor* , *Chenopodium* *quinoa* , *Tetragonia* *expansa* , *Nicotiana tabacum* var. *Xanthi-nc*, *Nicotiana* *glutinosa* , and *Nicotiana* *benthamiana* ) were from the CAU State Key Laboratory for Agrobiotechnology.

### RNA extraction, cloning and sequence analysis

Total RNA was extracted from potato plants collected in Hohhot (Inner Mongolia) and Qujin (Yunnan Province) using the published SDS–phenol/chloroform procedure [28] and it was used for reverse transcription (RT) using Moloney murine leukemia virus (M-MLV) reverse transcriptase (Promega, Madison, USA) according to the manufacturer’s instructions. Synthesized cDNA was amplified using LA *Taq* DNA polymerase (TaKaRa, Shiga, Japan). Rapid amplification of cDNA ends (RACE) was used to determine the 5' end of the viral genome using 5´ RACE System for Rapid Amplification of cDNA Ends, Version 2.0 (Invitrogen, Carlsbad, CA), according to the manufacturer’s instructions. The primers used in this study are shown in Table 1. A partial sequence of the new virus was obtained serendipitously during the detection of PLRV using the degenerate RT primer PoleR5 (and the PCR primers PL001F and PLN1674R). Degenerate primers were used to obtain a sequence upstream of this region, which were derived from an alignment of highly conserved regions of different carlaviral RNA sequences. To obtain the 3' sequence, the forward primer was designed based on the known sequence and used in combination with the Oligo (dT) primer HC511-18TR. The amplified products were purified from agarose gels using an AxyPrep^TM^ DNA Gel Extraction Kit (Axygen, Union City, CA), according to the manufacturer’s instructions, before they were ligated to the pMD19-T vector (TaKaRa, Shiga, Japan) and cloned into *Escherichia coli* MC1022 competent cells. Positive clones were identified by colony PCR.

**Table 1:** Polymerase chain reaction primers used for the amplification and sequencing of the PVH genome.

Primer name	Sequence (5' to 3')	Nucleotide position	Polarity	Method
PoleR5	CYCAKYTCCATSWYYC		Antisense	Reverse transcription
PL001F	ACAAAAGAATACCAGGAGGAATTGC		Sense	
PLN1674R	GACTTGTGGAGGCTTGTGAAAG		Antisense	
Carla114F	ATGGCWYTNACWTAYAGRASKCC	092-114nt	Sense	Degenerate PCR
Carla900F	GGACGATGGGCAGGTGTAC	855-873nt	Sense	
Car1340F	GTGGCGCTTGATAGGAAATC	1468-1487nt	Sense	
Carla1763F	GCCCGTTACAGTCGCGCCAG	1756-1775nt	Sense	
Carla3357R	GCTAATTTCGTAGACCAGAGAG	3336-3357nt	Antisense	Specific primer
Carla3226F	CTCGAGTACCTAGAGAGGAAGAG	3226-3248nt	Sense	Specific primer
Carla3600R	GTAGTTCGATGCCTCATGC	4839-4857nt	Antisense	
Carla4211R	CTATTCTCGAATTTTGGGTTG	3954-3974nt	Antisense	
Carla4610F	CAAGGTGGGTGCACAAAATC	4604-4623nt	Sense	
Carla5040R	CTATATCCACTAACCAATCCCTGC	5006-5029nt	Antisense	
Carla5468F	CCATGGCCAATATGCTTTTCAC	5468-5489nt	Sense	Specific primer
Carla5672R	CCATCAGCGCATAAGTTCCACC	5651-5672nt	Antisense	Specific primer
Carla5800F	CAGATTACACTTGCAACAGAAG	5029-5050nt	Sense	
Carla6670F	GGCTGACGACAAGAAGGG	7170-7187nt	Sense	
HC511-18TR	GG A T A T CTGCAG G A T C CAAGC(T)_18_	PolyA	Antisense	Reverse transcription
HC511R	GG A T A T CTGCAG G A T C CAAGC		Antisense	Anchored primer

^a^ The underlined sequences are the restriction sites specific for *Eco*RV (GAT↓ ATC) and *Bam*HI (G↓ GATCC). Protection bases are included in the 5' termini.

Sequences were analyzed using DNAMAN Version 5.2 (LynnonBiosoft, Quebec, Canada). BLASTx were used to compare each predicted PVH gene sequence with the non-redundant proteins database [29]. Sequences were aligned using Bioedit version 7.0.4.1[30]. PVH-Ho was used to explore the genome organization of PVH. Phylogenetic and molecular evolutionary analyses were conducted using MEGA version 4.1 [31]. Multiple alignments of protein-coding sequences were obtained using the default options in Clustal W [32]. Phylogenetic trees were constructed based on the aligned protein-coding sequences using the neighbor-joining method with a bootstrap value of 10000 replicates.

### Prokaryotic expression and antibody production

To facilitate prokaryotic expression, the PVH CP gene was amplified from the RT product by PCR and sub-cloned into the *Bam*HI and *Hin*dIII sites of the pET-28a (+) vector (Novagen Company, USA) with a his_6_ tag. The constructed recombinant plasmid pET-CP^PVH^ was then introduced into *Escherichia coli* BL21 (DE3) competent cells. PCR-verified positive clone cells were cultured at 37°C overnight, then inoculated into 1 L of fresh LB medium containing 50 μg/mL kanamycin before continuous culturing at 37°C for 4 h. Isopropyl-1-thio-Dgalactopyranoside (IPTG) was added to a final concentration of 0.3 mM to induce the expression of the his-tag fusion protein at 30°C overnight. Cells were harvested by centrifugation at 8,000 rpm for 10 min and resuspended in 100 mL PBS/urea buffer (50 mM phosphate-buffered saline buffer containing 8 M urea, pH 7.4) and sonicated on ice (3.0 s pulse with 3.0 s intervals for 9 min) using a Fisher Model 500 ultrasonic dismembrator (Fisher Scientific Co., Pittsburgh, PA, USA) in the presence of 1 mM DL-dithiothreitol (DTT). Further centrifugation (12,000 × g, 45 min, 4°C) and filtration on a membrane filter (0.45 μm) were used to remove any cell debris. The supernatant was then applied to a Ni-NTA agarose resin (Qiagen, Germany). After washing with five column-volumes of 10 mM imidazole in PBS/urea and five column-volumes of 20 mM imidazole in PBS/urea, the fusion protein was eluted from the column using 200 mM imidazole in PBS/urea, followed by dialysis and concentration. After prokaryotic expression and Ni-NTA resin purification, CP^PVH^ was prepared to raise the polyclonal antiserum in a New Zealand rabbit (prepared by the Institute of Genetics and Developmental Biology at the Chinese Academy of Sciences). Antiserum dilutions of 1:1000 and 1:2000 were used to test the polyclonal antiserum with the western blotting method. Both methods performed well at detecting the PVH-infected leaves of *Nicotiana* *glutinosa* .

Similar procedures were used to express the glutathione S-transferase (GST) tagged PVM and PVS CP genes, which were amplified and sub-cloned into the *Nco*I and *Hin*dIII sites of pGEX-KG, a prokaryotic expression vector with a GST tag. The purification method for GST-tagged proteins has been performed as described previously [33].

### Western blots

The purified CP^PVH^, CP^PVS^, and CP^PVM^ products, and the lyophilized positive control for PoLV (Agdia, Indiana USA) were resolved on 12.5% SDS-PAGE and electro-blotted onto HYBONDTM-C EXTRA membranes (Amersham, USA) using a Mini Trans-Blot electrophoretic transfer cell (Bio-Rad, USA) at 200 mA for 1.5 h. Nonspecific protein binding was blocked by incubating the membranes in PBST (PBS containing 0.05% Tween 20) containing 5% skim milk at 37°C for 1 h. The blots were incubated for 1 h at 37°C in PBST containing rabbit anti-CP^PVH^ polyclonal antiserum (1:1000 dilution) or detection antibodies against PVS, PVM, and PoLV (1:200 dilution) (Agdia, Indiana USA). After washing three times with PBST (10 min each), the proteins were incubated with alkaline phosphatase conjugated anti-rabbit IgG (from goat) (1:5000 dilution) (Sigma, USA) for 1 h (the blots incubated with the detection antibodies provided by Agdia skipped this step). Signals were detected using the colorimetric detection reagents nitro-blue tetrazolium chloride/5-bromo-4 -chloro-3-indolylphosphate (NBT/ BCIP).

### Electron microscopy

Partially purified PVH virus particles from infected *Nicotiana* *glutinosa* leaves were obtained using a modified protocol for PVS and PVM [34], and were adsorbed for 5 min onto Formvar-coated 200-mesh copper grids and negatively stained with 1% uranyl acetate (three times, 3 min per time) at room temperature (25°C). Digital images were captured using a transmission electron microscope (80KV, HITACHI, Ibaraki, Japan). The lengths of 103 particles were measured in the photomicrographs and analyzed using EXCEL.

### Inoculation assays

Indicator plants ( *Chenopodium* *amaranticolor* , *Chenopodium* *quinoa* , *Tetragonia* *expansa* , *Nicotiana tabacum* var. *Xanthi-nc*, *Nicotiana* *glutinosa* and *Nicotiana* *benthamiana* ) were germinated from seeds or propagated from seedlings (*Solanum tuberosum* and *S*. *lycopersicum*) grown from virus-free plantlets and maintained at 24°C with a 13-h (~75 µmol/m^2^.s) daylight regimen. Inocula were prepared by extracting the sap from PVH-infected potato leaves. The sap was diluted 1:5 with double-distilled water (ddH_2_O). The inoculation procedure involved rubbing the inocula mechanically onto the leaves of indicator plants and potato seedlings, which had been dusted with 600 mesh carborundum. Six to twelve plants were inoculated per species each time and the process was repeated three times. From 7 to 30 days post-inoculation (dpi), the inoculated leaves or upper leaves were collected to determine the presence of viruses by RT-PCR and western blotting.

The binary vector containing the full-length PVX (pPVX) was kindly provided by D. C. Baulcombe [35]. The CRP gene of PVH was amplified and subcloned into pPVX to synthesize the recombinant pPVX-pvhCRP. P0 of Brassica yellows virus (BrYV) [36], which was subcloned into pPVX to construct the positive control pPVX-P0. To inoculate the PVX recombinants, *Agrobacterium tumefaciens* strain GV3101 carrying the plasmids were agro-inoculated onto the 3- to 4-week-old *Nicotiana* *benthamiana* leaves. The agroinfiltration solution was infiltrated into the leaves to serve as a mock and pPVX-P0 was used as the positive control. Plants were photographed using a digital camera (550D, Canon, Japan) at 6 dpi and 12 dpi.

### GFP transient co-expression assay

PVH genes encoding TGB1 and CRP were cloned into the binary vector pGD [2] to construct the transient expression plasmids pGD-TGB1 and pGD-CRP. The empty vector pGD and pGD-HcPro, which was cloned from TEV, were used as the negative control and positive control, respectively. The recombinant *Agrobacterium tumefaciens* strain EHA105 was grown at 28°C in 2 mL of LB medium with 25 μg/mL rifampicin and 100 μg/mL kanamycin for 24 h, then transferred to 3 mL of fresh LB medium with appropriate antibiotics, before continuous overnight culture at 28°C. The bacteria were centrifuged at 5000 × g for 5 min. The pellet was resuspended in agroinfiltration solution (10 mM MgCl_2_, 10 mM MES and 100 μM acetosyringone). After analysis using a spectrophotometer, the optical density (OD_600_) of the culture was adjusted to 1.0. Equal volumes of culture (OD_600_=1.0) were mixed to facilitate co-infiltration with *A. tumefaciens* harboring pGD-GFP. The bacteria solution was kept at room temperature for at least 3 h before infiltration. Infiltration was performed using the underside leaves of *N* *. benthamiana* plants. After 3 or 5 dpi, the plants were observed to confirm silencing effect. The visual detection of GFP fluorescence in the infiltrated leaves was performed using a BLAK-RAY Non-UV semiconductor inspection lamp (B-100AP/R, UVP Inc., CA, USA). The plants were photographed using a digital camera (CoolPix 4500, Nikon, Japan) with a yellow filter (Kodak Wratten gelatin filter, no. 15,). The infiltrated area was collected separately and the area containing GFP was analyzed by western blotting using antiserum against GFP. To quantify the protein, Coomassie brilliant blue R250 was used (0.1% in 50% methanol/12% acetic acid) to stain the gel overnight with gentle shaking.

## Results

### A survey of potato for viruses revealed the existence of a novel carlavirus in China

The discovery of PVH was made fortuitously during a study of the distribution of potato viruses in China. During the detection of PLRV using RT-PCR with degenerate primers for poleroviruses, a much larger PCR product designated p1 (~3.4 kb rather than expected 1.6 kb) was obtained. BLAST search results showed that the generated fragment (p1) shared a homology at the nucleic acid level with the polymerase sequences of several members of the genus *Carlavirus* in the family *Betaflexiviridae*. However, the sequence identities shared between p1 and previously characterized carlaviruses were low (< 43%). This result revealed the existence of a new species in the genus *Carlavirus*. The virus is named tentatively PVH after Hohhot City in the Inner Mongolia Autonomous Region where the positive sample was collected.

Obtaining a PCR product from family *Betaflexiviridae* using degenerated and specific primers for poleroviruses is unexpected. During further analysis of the terminal sequence feature, one combination of the degenerate primers for PoleR5 (5'-CGCGGATCCATGTACC-3') was found to partially complement nt 7279–7293, while PL001F partially hybridized nt 3242–3276 of a novel (PVH) genome sequence.

### The genome structure and molecular characterization of PVH shows properties typical of the genus *Carlavirus*

**Figure 1:**
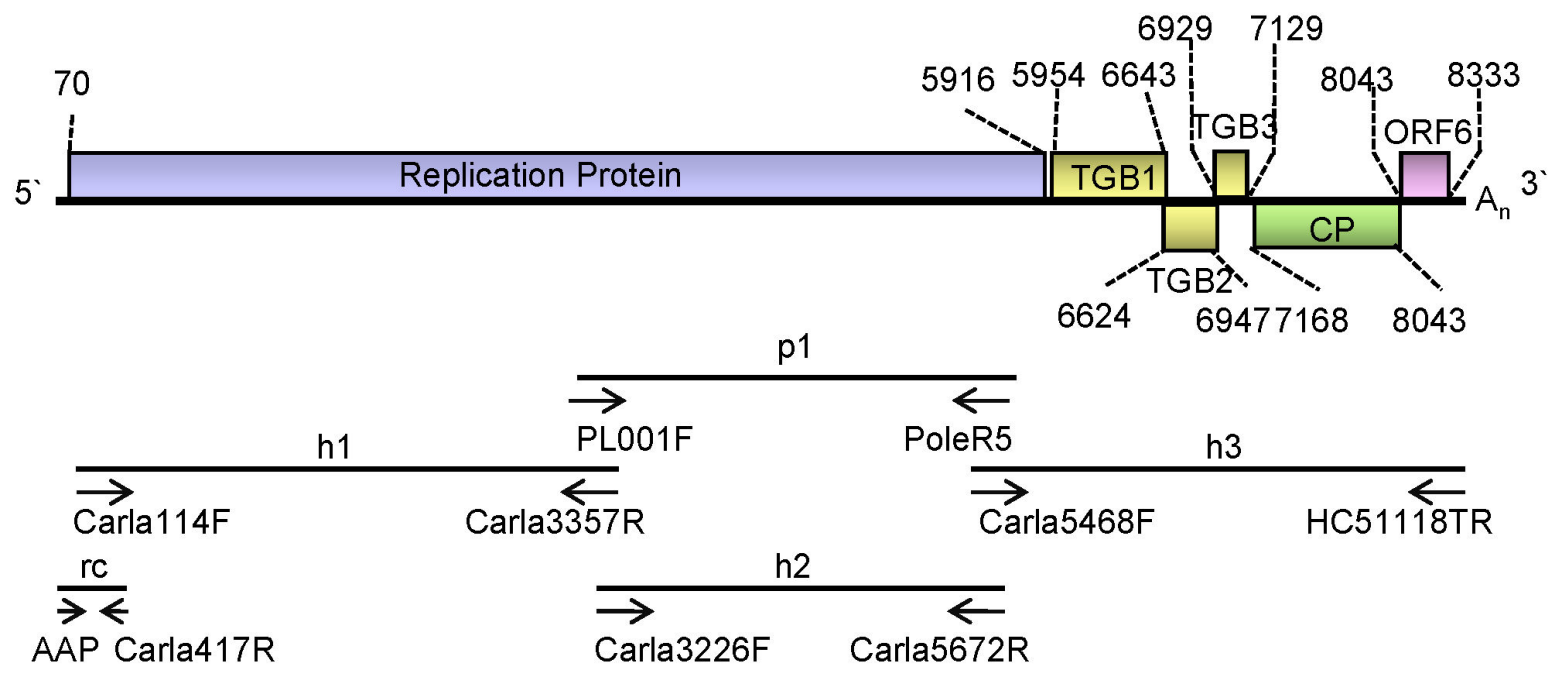
Genomic organization of PVH. (A) Schematic diagram of the PVH genome. The solid line represents the RNA genome and the boxes represent the ORFs. The putative protein products are indicated. (B) Genome cloning strategy and locations of the cDNA clones used for PVH sequencing: p1 represents the RT-PCR-generated sequence using the degenerate primer during the detection of *Potato leafroll virus*; h1, h2 and h3 represent RT-PCR-generated sequences using degenerate primers specific to carlaviruses and PVH specific primers based on the p1 sequence; rc is the 5-terminal clone generated by 5' rapid amplification of cDNA ends. The sequences of the primers are shown in Table 1.

The complete nucleotide sequences of the PVH genome from the Inner Mongolian isolate (designated PVH-Ho) and the Yunnan isolate (designated PVH-YN), were determined and submitted to GenBank (GenBank Accession No. HM584819 and JQ904630, respectively). The cloning strategy and a schematic representation of the PVH genome organization are shown in Figure 1. Table 1 shows the primers used applied to amplify the five overlapping PCR fragments, which covered the full viral genomic sequence. The 5' sequence (rc) was determined by 5' RACE using the abridged anchor primer (AAP) provided with the kit and two specific reverse primers. We sequenced at least three independent clones of each fragment and five independent clones of the 5' terminal fragment.

Sequence analysis showed that PVH-YN shared the same genome organization as PVH-Ho. The total length of the PVH-Ho and PVH-YN genomic RNA sequence was 8410 nt, excluding the poly (A) tails. The nucleotide sequence identity shared between the full-length genomes of the two PVH Chinese isolates was up to 93.58%. The PVH genome harbors six adjacent or overlapping open reading frames (ORFs). The 5' proximal ORF starts at nt 70 and extends to nt 5916. It encodes a replicase polyprotein with a predicted molecular mass of 219.62 kDa. Based on an analysis of the protein-coding sequence using the Conserved Domains Database on the NCBI website (http://www.ncbi.nlm.nih.gov/Structure/cdd/wrpsb.cgi) [37], five domains of PVH replicase had conserved orders that were typical of carlaviruses: viral methyltransferase (MTR), OTU-like cysteine protease, carlavirus endopeptidase, Superfamily 1 viral RNA helicase, and RNA dependent RNA polymerase. AlkB homology domains are present in the replicases of some carlaviruses (BlScV and PopMV) [38] but were not found in PVH replicase. There are three overlapping ORFs downstream of ORF1, i.e. ORF2 from nt 5954 to 6643, ORF3 from nt 6624 to 6947, and ORF4 from nt 6929 to 7129, which encode putative 25.77 kDa, 11.54 kDa, and 7.19 kDa proteins, respectively. These three proteins are believed to form a TGB. ORF 5 (nt 7168-8043) encodes the putative CP with a predicted molecular weight of 32.34 kDa. The 3' proximal ORF that encodes a 10.87 kDa CRP starts at nt 8043 and terminates at nt 8333, and encodes a putative nucleic acid-binding protein. All carlavirus CRPs contain a basic motif and a zinc finger-like motif in the central part of the protein [39,40]. These CRPs are predicted to have moderate sequence similarity to some cellular nucleic acid-binding proteins and PVM CRP is known to be a nucleic acid binding protein. The amino acid sequence analysis identified a zinc finger cysteine motif (C-X_2_-C-X_9_-C-X_4_-C) in the CRP of PVH. In addition, the 5' RACE analysis showed that the genome contains an UTR of 69 nt at the 5' terminus, and the 3' UTR of PVH was 74 nt in length, excluding the poly (A) tail. These features are similar to other species of the genus *Carlavirus*.

### Phylogenetic analysis of PVH and sequence comparisons with other carlaviruses confirms that it is a novel carlavirus

Compared with other carlaviruses, PVH-Ho was most similar to PoLV with the highest identity of 54.0%. The lowest similarity was to Apple stem pitting virus (ASPV), with an identity of 42.4%. ORF1 of PVH-Ho, which encodes a replicase protein, shared nucleotide identities of 49.8–54.0% and amino acid identities of 19.1–47.3% with other carlaviruses. The highest level of identity was also with PoLV, whereas the lowest level was with Sweet potato chlorotic fleck virus (SPCFV). Analysis of ORF5, which encodes CP, showed that the highest levels of nucleotide identity (54.0%) and amino acid identity (56.9%) were with *Hop latent virus* (HpLV) and Phlox virus M (PhVM), respectively, whereas the lowest levels of nucleotide identity (31.1%) and amino acid identity (17.9%) were with ASPV (Table 2).

**Table 2:** Sequence identity comparison of PVH-Ho and other carlaviruses.

	PVH-YN		Other carlaviruses	
	nt (%)	aa (%)	nt (%)	aa (%)
Full length	93.4		42.4-54.0	
5' UTR	94.3		46.5-61.3	
ORF1 (RdRp)	93.1	95.8	49.8-54.0	19.1-47.3
ORF2 (TGB1)	94.5	97.4	43.0-60.8	32.3-62.2
ORF3 (TGB2)	93.6	96.3	47.9-56.9	38.0-53.7
ORF4 (TGB3)	94.6	94.0	40.4-48.8	20.0-42.0
ORF5 (CP)	95.3	98.6	31.1-54.0	17.9-56.9
ORF6 (CRP)	94.2	93.8	29.3-43.5	15.4-37.6
3' UTR	100		58.75-66.2	

Note: PVH-Ho, Potato virus H from Hohhot in Inner Mongolia; PVH-YN, Potato virus H from Yunnan province.

**Figure 2:**
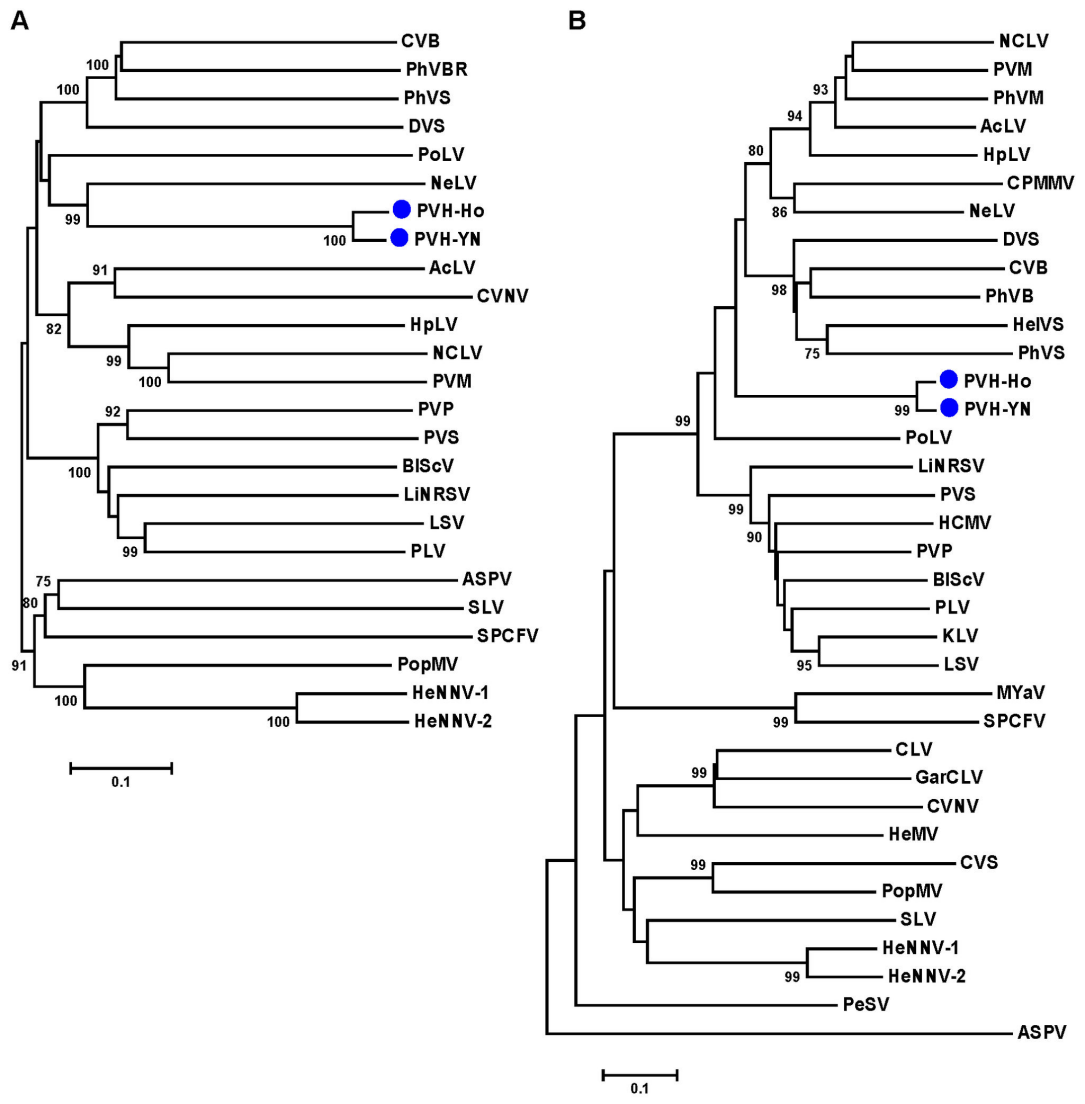
Phylogenetic analysis of PVH. Phylogenetic relationships of PVH and viruses in the genus *Carlavirus* within the family *Betaflexiviridae* based on the protein-coding sequences of (A) the replication polyprotein and (B) the coat protein. The neighbor-joining trees were constructed with maximum composite likelihood substitution model using MEGA (version 4.1). Bootstrap values shown at the branching points indicate the percentages of 10000 replications produced the clade (bootstrap values shown when >75%). Virus species and GenBank accession numbers used in the analysis: *Aconitum latent virus* (AcLV, AB051848); Apple stem pitting virus (ASPV, D21829); Blueberry scorch virus (BlScV, L25658); *Carnation latent virus* (CLV, AJ010697); *Cowpea mild mottle virus* (CPMMV, AF024628); *Chrysanthemum virus B* (CVB, AB245142); *Coleus vein necrosis virus* (CVNV, EF527260); Carrot carlavirus (CVS, EU881919); *Daphne virus S* (DVS, AJ620300); *Garlic common latent virus* (GarCLV, AB004566); Helleborus mosaic virus (HeMV, FJ196838); *Helleborus net necrosis virus-1* (HeNNV-1, FJ196835); *Helleborus net necrosis virus-2* (HeNNV-2, FJ196836); *Hop latent virus* (HpLV, AB032469); *Helenium virus S* (HelVS, D10454); *Hydrangea chlorotic mottle virus* (HCMV, DQ412999); *Kalanchoe latent virus* (KLV, AJ293570); *Ligustrum necrotic ringspot virus* (LiNRSV, EU074853); *Lily symptomless virus* (LSV, AJ564638); *Melon yellowing-associated virus* (MYaV, AY373028); *Narcissus common latent virus* (NCLV, AM158439); *Nerine latent virus* (NeLV, DQ098905); *Pea streak virus* (PeSV, AF354652); Phlox virus B (PhVB, EU162589); Phlox virus M (PhVM, EF507476); Phlox virus S (PhVS, EF492068); *Passiflora latent virus* (PLV, DQ455582); Poplar mosaic virus (PopMV, AY505475); *Potato latent virus* (PoLV, EU433397); Potato virus M (PVM, AJ437481); *Potato rough dwarf virus* or *Potato virus P* (PRDV or PVP, EU020009); Potato virus S (PVS, AJ863509); Shallot latent virus (SLV, AJ292226); Sweet potato chlorotic fleck virus (SPCFV, AY461421).

To further understand the molecular relationships between PVH and other reported carlaviruses, we constructed phylogenetic trees using CLUSTALW and they were visualized with MEGA (version 4.1). Phylogenetic analysis of the putative coding-sequence of PVH replicase and those of selected carlaviruses indicated that the two PVH isolates (PVH-Ho and PVH-YN) clustered together and formed a distinct branch (Figure 2A). They both clustered closer to *Nerine* *latent* *virus* (NeLV) than other species in the genus. In the phylogenetic tree based on the coding sequence of carlaviruses CPs, PVH-Ho and PVH-YN clustered together and formed a separate branch. This contrasted with the replicases, which were not close to other carlaviruses in the tree (Figure 2B).

### PVH was not related serologically to other potato carlaviruses

**Figure 3:**
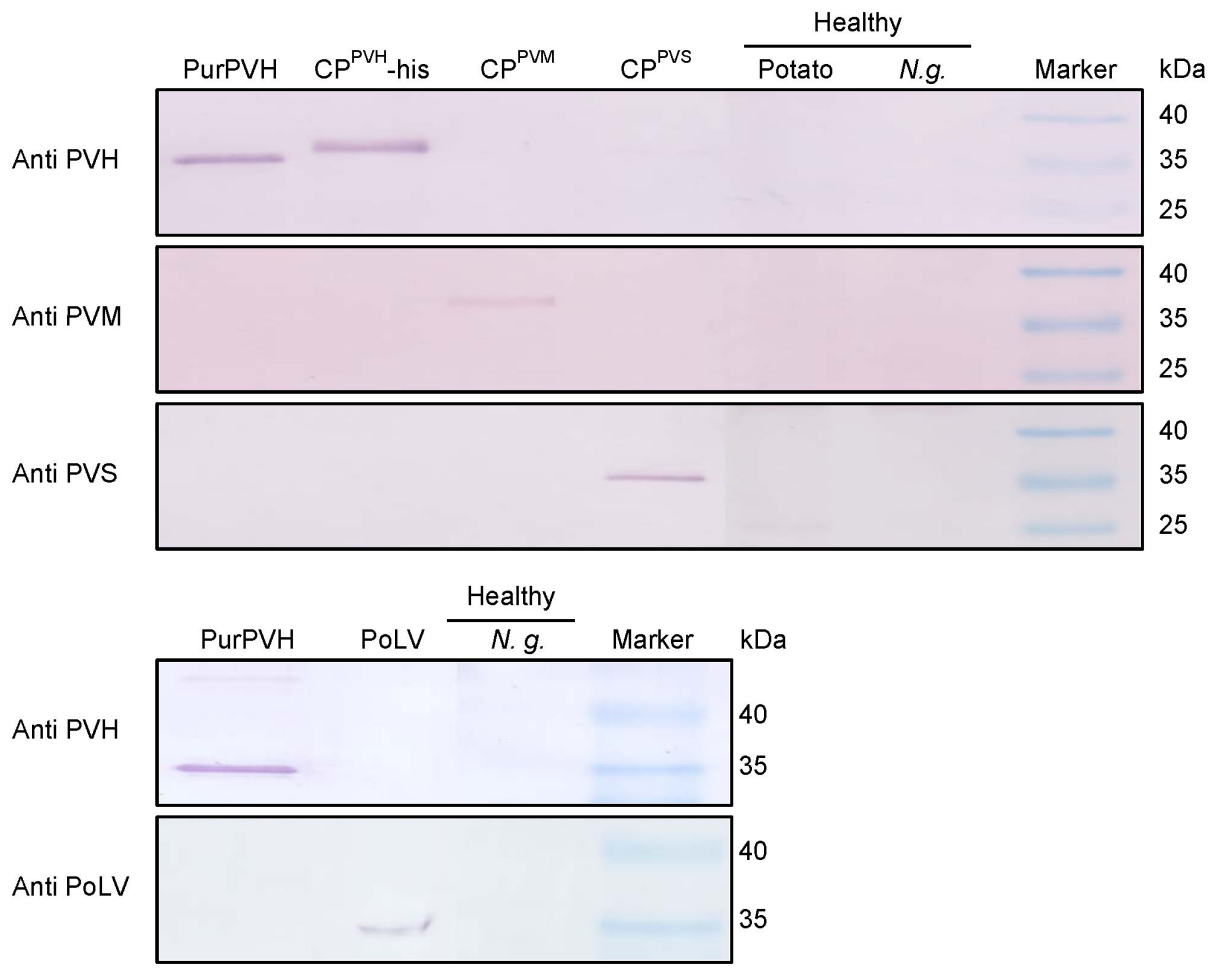
Serological relationships among different potato carlaviruses according to the western blotting analysis. The rabbit polyclonal antiserum raised against the coat protein (CP) of Potato virus H–Ho and the detection antibodies (Agdia, Indiana USA) against Potato virus M (PVM), Potato virus S (PVS), and *Potato latent virus* (PoLV) were used in the analysis. The purified PVH (PurPVH), purified pET-CP^PVH^ expression product (CP^PVH^-his), pGEX-CP^PVM^ expression product (CP^PVM^), pGEX-CP^PVS^ expression product (CP^PVS^), and the lyophilized positive control for PoLV (PoLV) (Agdia, Indiana USA) were used in the tests. Crude extracts from uninfected leaves of potato and *Nicotiana glutinosa *(*N. g.*) were used as the negative controls.

The serological relationships between PVH and other potato carlaviruses were determined using western blotting. The purified CP^PVH^-his prokaryotic expression product and virus purified from PVH-infected *N* *. glutinosa* leaf samples reacted strongly to the polyclonal antiserum against CP^PVH^, but not to the antibodies against PVS, PVM, or PoLV. The purified CP^PVS^, CP^PVM^, and positive control for PoLV reacted negatively to the polyclonal antiserum against CP^PVH^ (Figure 3). These results show that PVH is not related serologically to other potato carlaviruses (PVS, PVM, and PoLV).

### PVH virion morphology and distinct host range

**Figure 4:**
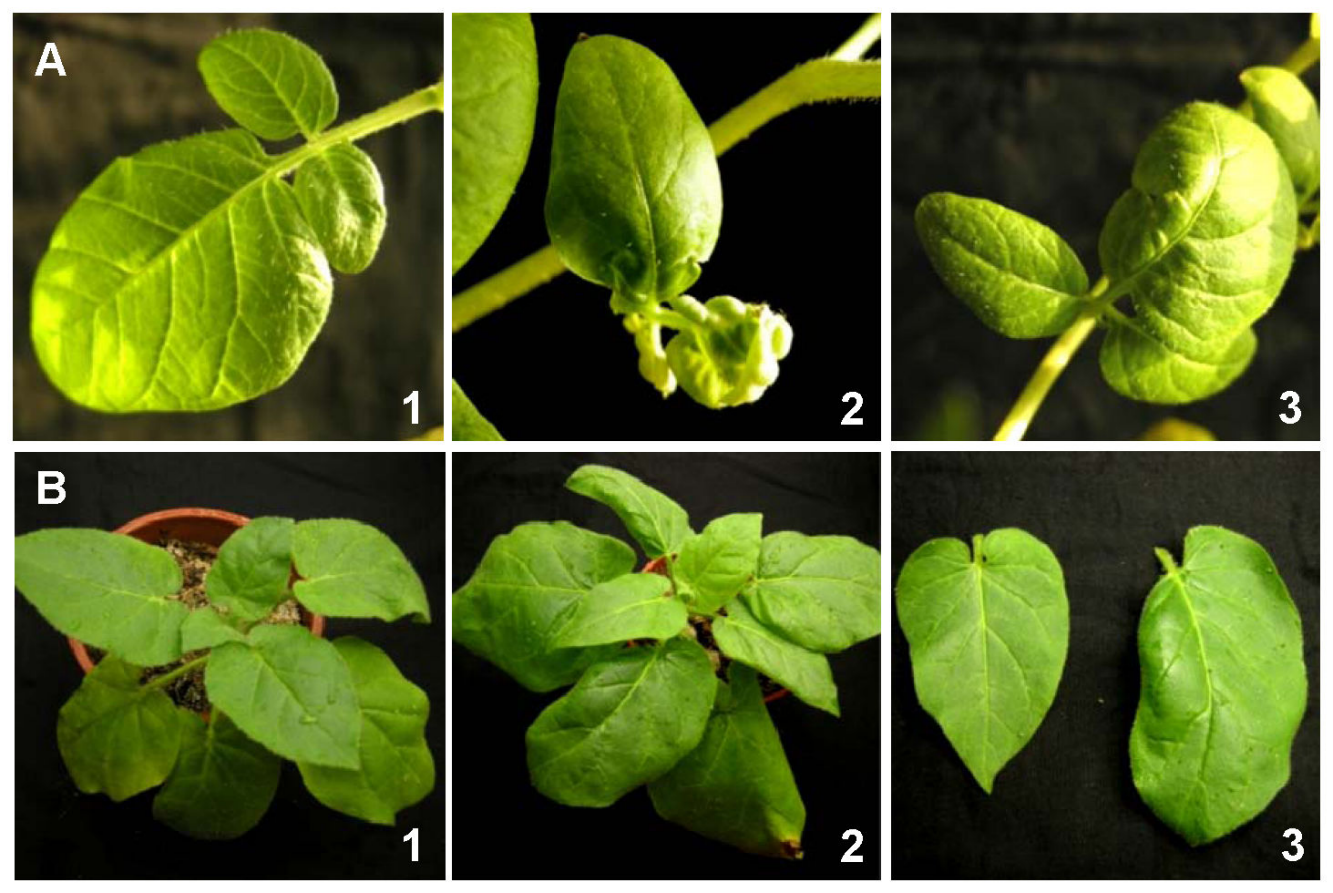
Systemic symptoms caused by PVH in potato cv. Shepody and *Nicotiana glutinosa* (*N. g.*) leaves. (A) Systemic effects on the leaves of potato cv. Shepody at 60 dpi. 1, leaf of mock Shepody plant inoculated with ddH_2_O; 2, 3, leaves of Shepody plant inoculated with PVH. (B) *N. g*. systemic leaves at 53 dpi. 1, Mock *N. g*. plant inoculated with ddH_2_O; 2, *N. g*. plant inoculated with PVH; 3, leaf of *N. g*. plant inoculated with ddH_2_O (left) and leaf of *N. g*. plant inoculated with PVH (right).

PVH appeared to have a host range that is very different from other potato carlaviruses (PVS^O^ [5,41], PoLV [5], PVM [42], and PVP [6]) (Table 3). Among the eight indicator and host plants included in this study, PVH could systemically infect *Nicotiana* *glutinosa* , *Solanum* *lycopersicum* , and potato, but not the other plants. PVH produced mild symptoms in the host plants. A slight leaf curl and dark green mottle were observed in PVH-infected *N* *. glutinosa* (Figure 4B), but not in potato (*S. tuberosum*, cv. Shepody), where only a slight leaf curl was observed (Figure 4A). Most PVH-infected plants remained symptomless or latent.

**Table 3:** Host ranges of PVH and other potato carlaviruses.

Host plant	PVH^a^	PVS^b^	PVM^c^	PoLV^b^	PVP^d^
*Solanum tuberosum*	S	S	S	S	S
*Solanum* *lycopersicum*	S	N	S	N	N
*Nicotiana* *glutinosa*	S	N	S	S	N
*Nicotiana* *benthamiana*	N	S	S	S	N
*Nicotiana tabacum*	N	N	N	S	N
*Chenopodium* *amaranticolor*	N	L	nd	S	nd
*Chenopodium* *quinoa*	N	L	nd	L	nd
*Tetragonia* *expansa*	N	nd	nd	nd	nd

S, systemic infection; L, local infection; N, not infected; nd, not detected

a results of RT-PCR and Western blotting 15–30 dpi; b results of enzyme-linked immunosorbent assay (ELISA) 2–4 weeks after inoculation [5] and triple antibody sandwich enzyme-linked immunosorbent assay (TAS-ELISA) [41]; c results of tissue blot immunosorbent assay (TBIA) from 17 to 52 dpi [42]; d results of double antibody sandwich enzyme-linked immunosorbent assay (DAS-ELISA) for differential hosts [6].

**Figure 5:**
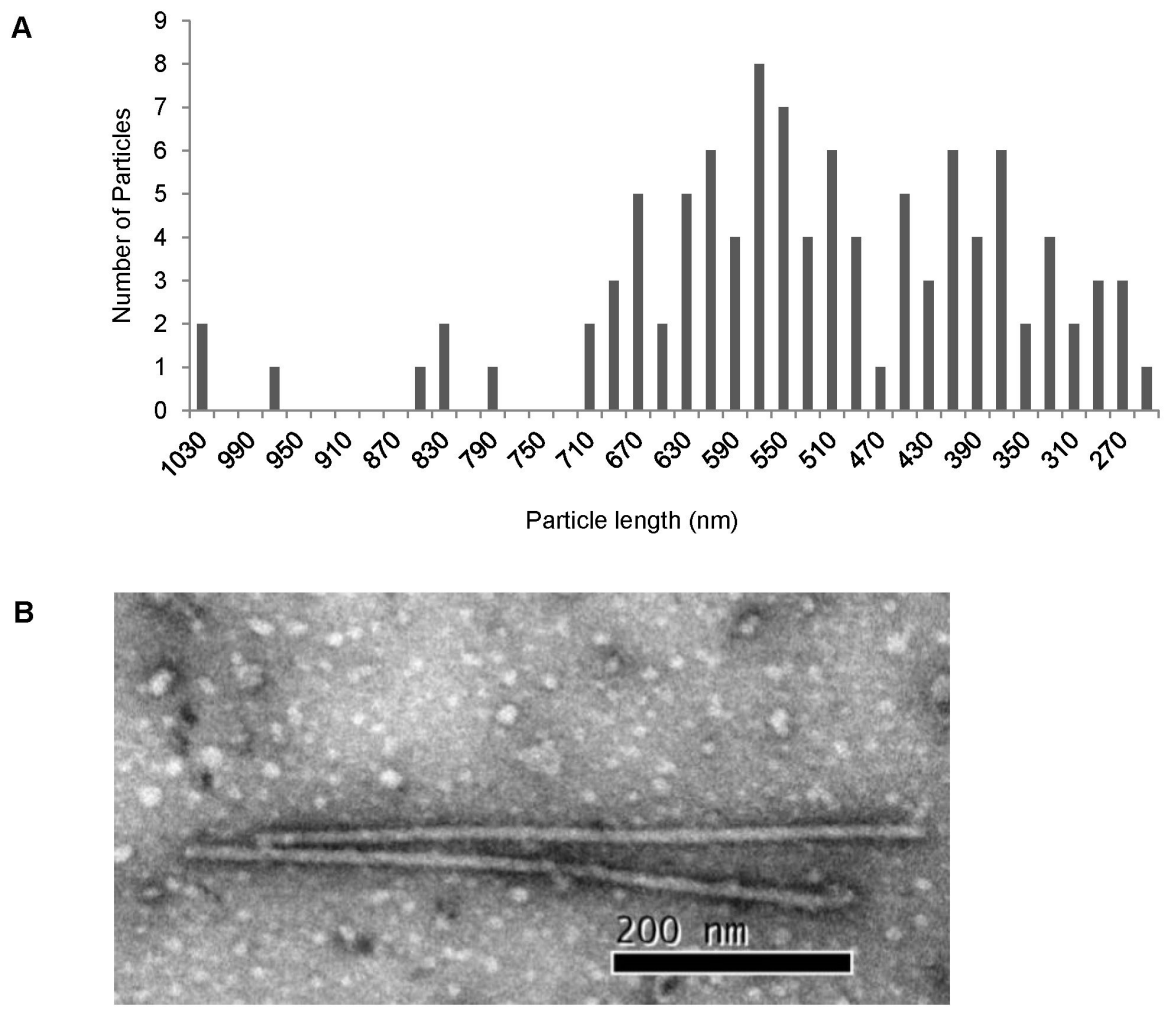
PVH viral particles and particle length distribution. (A) Particle length distribution of PVH. (B) Transmission electron micrograph showing the negative-stained virion of purified PVH.

Electron microscopy of the partially-purified PVH virus showed that the viral particles were filamentous and slightly curved (Figure 5B), with a modal length of 570 nm. The length of the PVH particles varied from 250 to 1030 nm, with a peak of 570 nm (Figure 5A).

### CRP suppresses local RNA silencing and enhances the pathogenicity of PVX

**Figure 6:**
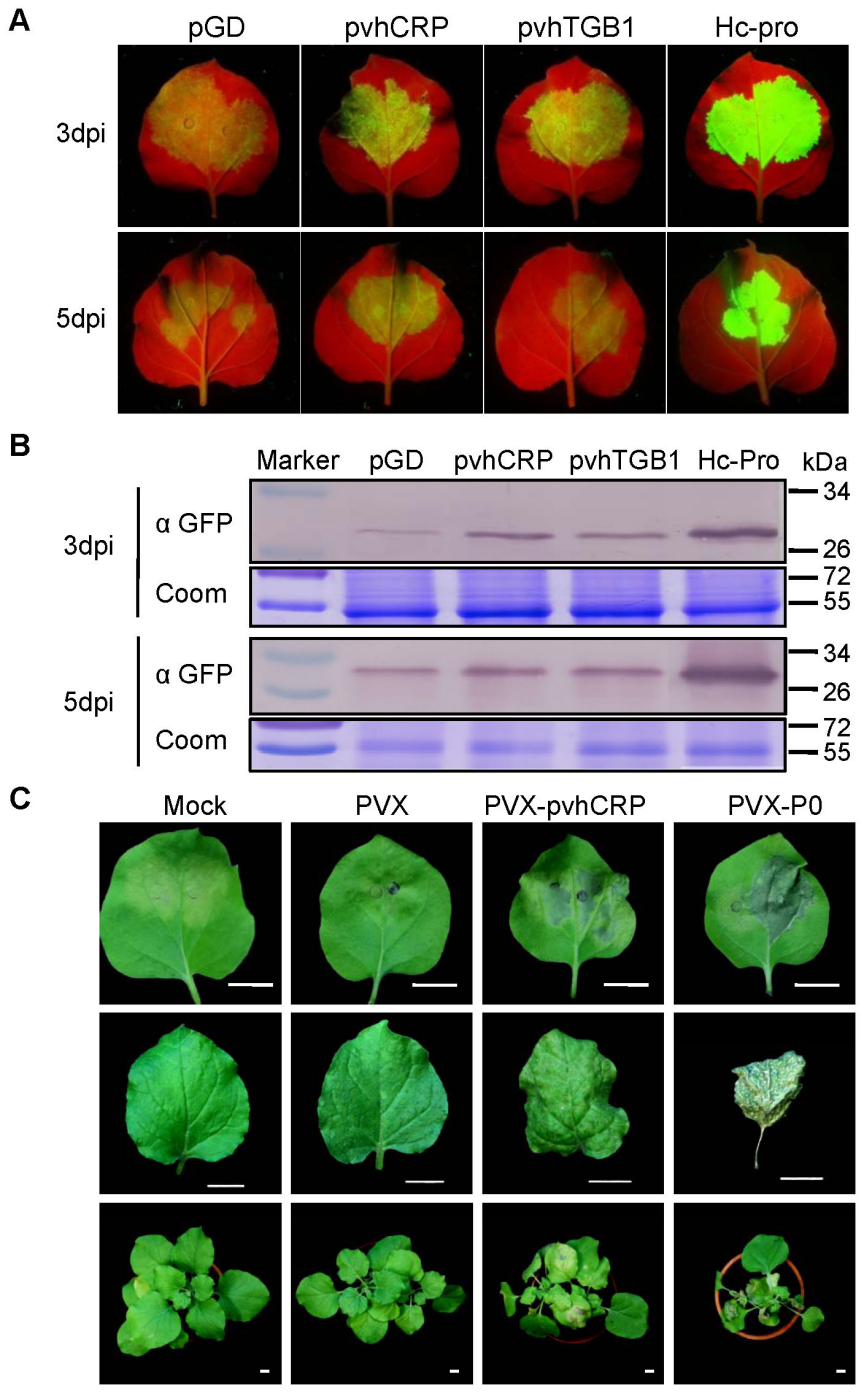
Suppressive activities of pvhCRP and pvhTGB1 on local RNA silencing. (A) GFP image of co-infiltrated *N. benthamiana* leaves under an UV lamp at 3 dpi and 5 dpi. (B) Standard western blotting analysis of GFP in co-infiltrated *N. benthamiana* leaves. coom, Coomassie brilliant blue-stain. (C) Symptoms in pPVX, pPVX-pvhCRP or pPVX-P0 infected *N. benthamiana* plants. The inoculated leaves (upper panels) were taken at 6 dpi. The systemically infected leaves (middle panels) and images of whole plants (lower panels) were at 12 dpi. Scale bar, 1 cm.

To determine whether the CRP and TGB1 of PVH had RNA silencing suppression activities, an *Agrobacterium tumefaciens* strain EHA105 that harbored GFP-expressing plasmid GD-GFP was mixed with another EHA105 that carried either a test or a control construct. The mixture was co-infiltrated into leaves of *N* *. benthamiana* wild-type (WT) and six plants were used for each treatment. The experiment was replicated three times. In Figure 6A, the green fluorescent protein transient co-expression assay showed that GFP expression was maintained in the patches co-infiltrated with pGD-CRP at 3 dpi and 5 dpi. By contrast, the GFP expression in the leaves co-infiltrated with pGD-TGB1 was maintained at 3 dpi but it was not different from the empty vector at 5 dpi. These results were confirmed by western blotting with GFP antiserum (Figure 6A).

Several viral silencing suppressors have been reported to cause more severe symptoms when they are expressed by a heterologous viral expression vector such as PVX [43,44]. In the recombinant PVX expression assay, the CRP of PVH enhanced the pathogenicity of recombinant PVX in *Nicotiana* *benthamiana* (Figure 6B). The CRP of PVH appears to act as a pathogenicity determinant and a suppressor of local RNA silencing.

### Distribution of PVH in China

Our investigation showed that PVH was present in most potato growing areas of China. PVH was detected readily in potato leaves and/or tubers using RT-PCR with the primers PVHCPF (5'-CGGGATCCATGGATGTTGCTACTTC-3') and PVHCPR (5'-CCAAGCTTATTGCCGCTTGTTATTC-3'). We found that 35/171 potato leaves and tuber samples collected from five provinces and the Inner Mongolia Autonomous region were infected with PVH. Of these, 22 samples were co-infected with PVH and PVS, PVX, PVY, PVM, and/or PLRV in various combinations (Table 4).

**Table 4:** Distribution and incidence of PVH in six potato-producing provinces in China.

Source of samples	No. of samples					
	Total	Positive for PVH^a^	PVH mixed infected with			
			PVS	PVX	PVY	PLRV
Inner Mongolia	57	9	3	4	3	3
Yunnan	54	21	5	10	2	0
Hebei	7	2	0	0	0	0
Heilongjiang	40	1	0	0	0	0
Xinjiang	8	1	1	1	0	0
Liaoning	5	1	1	1	1	1
Total surveyed	171	35	10	16	6	4

Note: Samples were detected by RT-PCR and/or western blotting.

## Discussion

The genus Carlavirus belongs to the newly established virus family *Betaflexiviridae*. According to the species demarcation criteria given in the Ninth Report of the International Committee on Taxonomy of Viruses (ICTV), distinct species in the genus *Carlavirus* should: a) have a specific natural host range; b) not cross-protect in infected common host plant species; c) be readily differentiated using serological procedures; and d) have less than about 72% shared nt identity (or 80% aa identity) in their CP or polymerase genes [2]. In the present study, we discovered a novel virus that infects potatoes in China. We determined the completed genomic sequence to investigate the molecular features and phylogenetic position of this virus. The genome sequence and predicted genome organization indicated that the virus shared similar properties with members of the genus *Carlavirus*, although the CP and replicase amino acid identities were only 17.9–56.9% and 19.1–47.3%, respectively, compared with those of reported carlaviruses. The phylogenetic analyses based on the complete polymerase and CP sequences indicated that, although this virus belongs to the genus *Carlavirus*, it is distinct from all recognized species (43 species) [2]. Moreover, this novel virus was unrelated serologically to other potato carlaviruses (PVS, PVM, and PoLV); it had a distinct host range (Table 3), and was capable of co-infecting potato plants with PVS, PVX, PVY, PVM, and PLRV. Negative staining of the partially purified virus identified the slightly curved filamentous virion particles, which are typical of carlaviruses. Based on the evidence presented and the ICTV guidelines, we propose the classification of this newly identified virus as the prototype member of Potato virus H (PVH), a new species in the genus *Carlavirus.*

RNA silencing is a defense mechanism employed by plants to protect against viruses and transposons [45-48]. As counter-defenses, viruses have developed diverse strategies to evade RNA-silencing immunity. Numerous RNA silencing suppressors have been identified in plant viruses [47,49], although very few have been identified in the genus *Carlavirus*. In the present study, we demonstrated that the CRP of PVH suppressed local RNA silencing during a GFP transient co-expression assay (Figure 6A) while it enhanced the pathogenicity of recombinant PVX. These results agreed with previous studies of PVM [13]. During the conventional GFP transient co-expression assay, the CRP of PVM exhibited suppressor activity for local and systemic silencing and it increased the accumulation of viral RNA at the single-cell level. TGBp1 of PVM did not inhibit local RNA silencing in the GFP transient co-expression assay but it was a systemic silencing suppressor and promoted viral cell-to-cell movement by inhibiting RNA silencing [13]. In Chrysanthemum virus B (CVB), the expression of the CRP (p12) gene in the PVX genome dramatically affected the symptoms produced by the virus in *N* *. benthamiana* , whereas p12 was unable to suppress the silencing induced locally by dsRNA or ssRNA [11]. Recently, the p12 protein encoded by CVB was shown to act as a plant transcription factor and an activator of the expression of a regulator of cell size and proliferation, which causes hyperplasia [50]. It is quite interesting that there are fundamental differences among the homologous proteins encoded by viruses in the same genus. Given that PVH infection also causes leaf malformation in *N* . *glutinosa* and potato cv. Shepody (Figure 4), it is possible that the CRP of PVH has the same function as the p12 of CVB.

Our studies indicated that PVH is widespread in China. PVH is readily transmitted mechanically and it was detected in seed tubers, which indicate a strong dissemination potential. In contrast to PVX and PLRV, the expressed symptoms during a sole infection with PVH were often latent, or a mild leaf curl and mottling in *Nicotiana* *glutinosa* and potato cv. Shepody. Like other carlaviruses such as PVS and PVM, PVH readily co-infects potatoes on its own or with other viruses. Our results showed that PVH could be present in mix-infected with PVS, PVX, PVY, PVM, or PLRV in various combinations. Potato plants co-infected with all six major viruses were found in Liaoning. Potatoes with co-infections of viruses had more severe symptoms and greater yield losses. For example, a PVS and PVX mixed infection increased the titer of PVS in plant tissues, enhanced the expression of foliar symptoms in primarily- and secondarily-infected plants of the variety ‘Royal Blue’, and reduced yield by 23% [51]. The changes in titer (increase) and yield (decrease) suggested that there was a synergistic relationship between PVS and PVX. In another study, a recombinant potexvirus was constructed using the TGB from PVS. This complementation produced a stable virus that acquired a new function, which facilitated a change of host range and aphid transmissibility [10]. Overall, these findings suggest that PVH and other potato viruses in co-infection may lead to higher levels of viral replication, more severe symptoms and even significant potato yield losses.

In conclusion, the complete genome sequence, and the serological and biological analyses demonstrate that PVH is a new, distinct carlavirus, which is widespread in potatoes in China. In general, PVH was present in co-infections with PVS and other potato viruses. The origin of this virus and its worldwide distribution, its pathogenic vector transmission, and the effects of co-infection with other carlaviruses or important potato viruses such as PVX and PLRV still need to be elucidated.

## References

[1] Roger ACJ, Amy C, Cesar EF, Walter RS, Steven AS (2009) Potato Virus and Viruslike Diseases. In: Virus Diseases of Plants: Grape, Potato, and Wheat Image Collection and Teaching Resource CD-Rom. St. Paul, MN: APS Press.

[2] King A, Lefkowitz E, Adams MJ (2011) Virus Taxonomy: Ninth Report of the International Committee on Taxonomy of Viruses: An Elsevier Title.

[3] de Bruyn Ouboter MP (1951) A new potato virus. Proc 1st Conf Potato Virus Diseases. Lisse-Wageningen pp. 83.

[4] Schultz E, Folsom D (1923) Transmission, variation, and control of certain degeneration diseases of Irish potatoes. Agric Res 25: 393-396.

[5] Brattey C, Badge JL, Burns R, Foster GD, George E, et al. (2002) Potato latent virus: a proposed new species in the genus Carlavirus. Plant Pathol 51: 495-505. doi:10.1046/j.1365-3059.2002.00729.x.

[6] Massa GA, Segretin ME, Colavita M, Riero MF, Bravo-Almonacid F, et al. (2006) Biological and sequence data suggest that potato rough dwarf virus (PRDV) and potato virus P (PVP) are strains of the same species. Arch Virol 151: 1243-1247. doi:10.1007/s00705-006-0760-9. PubMed: 16601924. 16601924 10.1007/s00705-006-0760-9

[7] Nisbet C, Butzonitch I, Colavita M, Daniels J, Martin J, et al. (2006) Characterization of Potato rough dwarf virus and Potato virus P: distinct strains of the same viral species in the genus Carlavirus. Plant Pathol 55: 803-812. doi:10.1111/j.1365-3059.2006.01448.x.

[8] Nie XZ, Bai YJ, Molen TA, Desjardins DC (2008) Development of universal primers for detection of potato carlaviruses by RT-PCR. J Virol Methods 149: 209-216. doi:10.1016/j.jviromet.2008.02.004. PubMed: 18353450. 18353450 10.1016/j.jviromet.2008.02.004

[9] Salazar LF (1996) Potato viruses and their control. Lima: International Potato Center (CIP).

[10] Matousek J, Schubert J, Dedic P (2009) Complementation analysis of triple gene block of Potato virus S (PVS) revealed its capability to support systemic infection and aphid transmissibility of recombinant Potato virus X. Virus Res 146: 81-88. doi:10.1016/j.virusres.2009.09.003. PubMed: 19748533. 19748533 10.1016/j.virusres.2009.09.003

[11] Lukhovitskaia NI, Solov’eva AG, Koshkina TE, Zavriev SK, Morozov S (2005) Interaction of cysteine-rich protein of Carlavirus with plant defense system. Mol Biol (Mosk) 39: 896-904. 16240723

[12] Lukhovitskaya NI, Ignatovich IV, Savenkov EI, Schiemann J, Morozov SY, et al. (2009) Role of the zinc-finger and basic motifs of chrysanthemum virus B p12 protein in nucleic acid binding, protein localization and induction of a hypersensitive response upon expression from a viral vector. J Gen Virol 90: 723-733. doi:10.1099/vir.0.005025-0. PubMed: 19218219. 19218219 10.1099/vir.0.005025-0

[13] Senshu H, Yamaji Y, Minato N, Shiraishi T, Maejima K, et al. (2011) A dual strategy for the suppression of host antiviral silencing: two distinct suppressors for viral replication and viral movement encoded by potato virus M. J Virol 85: 10269–10278. doi:10.1128/JVI.05273-11. PubMed: 21752911. 21752911 10.1128/JVI.05273-11 PMC3196401

[14] Bode O, Weidemann H (1971) Untersuchungen zur Blattlausübertragbarkeit von Kartoffel-M-und-S-Virus. Potato Res 14: 119-129. doi:10.1007/BF02361820.

[15] MacKinnon J (1974) Detection, spread, and aphid transmission of potato virus S. Can J Bot 52: 461-465. doi:10.1139/b74-060.

[16] Kostiw M (1975) Transmission of potato virus S byAphis nasturtii Kalt. Potato Res 18: 641-643. doi:10.1007/BF02365690.

[17] Wetter C, Völk J (1960) Versuche zur Übertragung der Kartoffelviren M und S DurchMyzus Persicae Sulz. Potato Res 3: 158-163.

[18] Loebenstein G (2001) Virus and virus-like diseases of potatoes and production of seed-potatoes. Kluwer Academic Publishers: Dordrecht,The Netherlands.

[19] Vreugdenhil D (2007) Potato biology and biotechnology: advances and perspectives. Elsevier: Amsterdam, The Netherlands.

[20] Wang B, Ma Y, Zhang Z, Wu Z, Wu Y, et al. (2011) Potato viruses in China. Crop Protect 30: 1117-1123. doi:10.1016/j.cropro.2011.04.001.

[21] Bai YJ, Wen JZ, Yang MX, Yu DC, Gao YL, et al. (2007) Comparison of incidence of major potato viruses in southwest and northeast potato-producing regions in China. J Northeast Agri Uni 38: 733-736.

[22] Zhou QM, Xie XL, Wen CX, Ma H, Yin J, et al. (2007) Molecular Identification of Potato virus S Hebei Isolate. Acta Agriculturae BorealiSinica 22: 39-42.

[23] Zhong TT, Pu ZG, He JR, Wu J, Yang CL, et al. (2008) The latest investigation and serological detection of potato viruses in Sichuan province. Southwest China J Agricul Sci 21: 96-99.

[24] Huang P, Yan Q, Ding Y (2009). Occurrence and Control of Potato Virus S in Guizhou. Guizhou Agricul Sci 37: 88-89.

[25] Zhang W, Bai YQ, Gao YL, Sheng Y, Fan GQ, et al. (2010). Investigation on Virus Disease of the Potato Main Production Area. Heilongjiang Agri Sci 4: 71-73.

[26] Wang XM, Jin LP, Yin J (2005) Progress in Breeding for Virus Resistance in Potatoes. Chin Potato J 19: 285-289.

[27] Liu HY, Zhang HX, Li MF, Yang LQ, Teng LY et al (2006) The overall survey and identification of potato virus diseases in Heilongjiang Province. J Northeast Agri Uni 37: 307-310.

[28] Han C, Li D, Xing Y, Zhu K, Tian Z, et al. (2000) Wheat yellow mosaic virus Widely Occurring in Wheat (*Triticum aestivum*) in China. Plant Dis 84: 627-630. doi:10.1094/PDIS.2000.84.6.627. 10.1094/PDIS.2000.84.6.627 30841101

[29] Altschul SF, Madden TL, Schäffer AA, Zhang J, Zhang Z, et al. (1997) Gapped BLAST and PSI-BLAST: a new generation of protein database search programs. Nuc Acids Res 25: 3389-3402. doi:10.1093/nar/25.17.3389. PubMed: 9254694. 10.1093/nar/25.17.3389 PMC146917 9254694

[30] Hall TA (1999) BioEdit: a user-friendly biological sequence alignment editor and analysis program for Windows 95/98/NT. Nuc Acids Sympos Ser 41: 95-98.

[31] Tamura K, Dudley J, Nei M, Kumar S (2007) MEGA4: molecular evolutionary genetics analysis (MEGA) software version 4.0. Mol Biol Evol 24: 1596-1599. doi:10.1093/molbev/msm092. PubMed: 17488738. 17488738 10.1093/molbev/msm092

[32] Thompson JD, Higgins DG, Gibson TJ (1994) CLUSTAL W: improving the sensitivity of progressive multiple sequence alignment through sequence weighting, position-specific gap penalties and weight matrix choice. Nuc Acids Res 22: 4673-4680. doi:10.1093/nar/22.22.4673. PubMed: 7984417. 10.1093/nar/22.22.4673 PMC308517 7984417

[33] Harper S, Speicher DW (1999) Expression and purification of GST fusion proteins. Current Protocols in Protein Science. John Wiley and Sons: New York. 10.1002/0471140864.ps0606s09 18429193

[34] Tavantzis S (1983) Improved purification of two potato carlaviruses. Phytopathol 73: 190-194. doi:10.1094/Phyto-73-190.

[35] Baulcombe DC, Chapman S, Cruz S (2002) Jellyfish green fluorescent protein as a reporter for virus infections. Plant J 7: 1045-1053. PubMed: 7599646. 10.1046/j.1365-313x.1995.07061045.x 7599646

[36] Xiang HY, Dong SW, Shang QX, Zhou CJ, Li DW, et al. (2011) Molecular characterization of two genotypes of a new polerovirus infecting brassicas in China. Arch Virol 156: 2251-2255. doi:10.1007/s00705-011-1091-z. PubMed: 21874520. 21874520 10.1007/s00705-011-1091-z

[37] Marchler-Bauer A, Lu S, Anderson JB, Chitsaz F, Derbyshire MK, et al. (2011) CDD: a Conserved Domain Database for the functional annotation of proteins. NucAcids Res 39: D225-D229. 10.1093/nar/gkq1189 PMC3013737 21109532

[38] Martelli GP, Adams MJ, Kreuze JF, Dolja VV (2007) Family Flexiviridae: a case study in virion and genome plasticity. Annu Rev Phytopathol 45: 73-100. doi:10.1146/annurev.phyto.45.062806.094401. PubMed: 17362202. 17362202 10.1146/annurev.phyto.45.062806.094401

[39] Gramstat A, Courtpozanis A, Rohde W (1990) The 12 kDa protein of potato virus M displays properties of a nucleic acid-binding regulatory protein. FEBS Lett 276: 34-38. doi:10.1016/0014-5793(90)80500-I. PubMed: 2265707. 2265707 10.1016/0014-5793(90)80500-i

[40] Koonin EV, Boyko VP, Dolja VV (1991) Small cysteine-rich proteins of different groups of plant RNA viruses are related to different families of nucleic acid-binding proteins. Virol 181: 395-398. doi:10.1016/0042-6822(91)90512-A. PubMed: 1994589. 10.1016/0042-6822(91)90512-a 1994589

[41] Goth RW, Ellis PJ, de Villiers G, Goins EW, Wright NS (1999) Characteristics and distribution of potato latent carlavirus (Red LaSoda virus) in North America. Plant Dis 83: 751-753. doi:10.1094/PDIS.1999.83.8.751. 10.1094/PDIS.1999.83.8.751 30845562

[42] Flatken S, Ungewickell V, Menzel W, Maiss E (2008) Construction of an infectious full-length cDNA clone of potato virus M. Arch Virol 153: 1385-1389. doi:10.1007/s00705-008-0127-5. PubMed: 18543062. 18543062 10.1007/s00705-008-0127-5

[43] Brigneti G, Voinnet O, Li WX, Ji LH, Ding SW, et al. (1998) Viral pathogenicity determinants are suppressors of transgene silencing in Nicotiana benthamiana. The EMBO J 17: 6739-6746. 9822616 10.1093/emboj/17.22.6739 PMC1171019

[44] Pfeffer S, Dunoyer P, Heim F, Richards KE, Jonard G, et al. (2002) P0 of Beet western yellows virus is a suppressor of posttranscriptional gene silencing. J Virol 76: 6815–6824. doi:10.1128/JVI.76.13.6815-6824.2002. PubMed: 12050394. 12050394 10.1128/JVI.76.13.6815-6824.2002 PMC136274

[45] Baulcombe D (2004) RNA silencing in plants. Nature 431: 356-363. doi:10.1038/nature02874. PubMed: 15372043. 15372043 10.1038/nature02874

[46] Brodersen P, Voinnet O (2006) The diversity of RNA silencing pathways in plants. Trends Genet 22: 268-280. doi:10.1016/j.tig.2006.03.003. PubMed: 16567016. 16567016 10.1016/j.tig.2006.03.003

[47] Li F, Ding SW (2006) Virus counterdefense: diverse strategies for evading the RNA-silencing immunity. Annu Rev Microbiol 60: 503-531. doi:10.1146/annurev.micro.60.080805.142205. PubMed: 16768647. 16768647 10.1146/annurev.micro.60.080805.142205 PMC2693410

[48] Ding SW, Voinnet O (2007) Antiviral immunity directed by small RNAs. Cell 130: 413-426. doi:10.1016/j.cell.2007.07.039. PubMed: 17693253. 17693253 10.1016/j.cell.2007.07.039 PMC2703654

[49] Voinnet O (2005) Induction and suppression of RNA silencing: insights from viral infections. Nat Rev Genet 6: 206-220. doi:10.1038/nrg1555. PubMed: 15703763. 15703763 10.1038/nrg1555

[50] Lukhovitskaya NI, Solovieva AD, Boddeti SK, Thaduri S, Solovyev AG, et al. (2013) An RNA Virus-Encoded Zinc-Finger Protein Acts as a Plant Transcription Factor and Induces a Regulator of Cell Size and Proliferation in Two Tobacco Species. Plant Cell online. 10.1105/tpc.112.106476 PMC3634699 23482855

[51] Nyalugwe E, Wilson C, Coutts BA, Jones R (2012) Biological properties of Potato virus X in potato: effects of mixed infection with Potato virus S and resistance phenotypes in cultivars from three continents. Plant Dis 96: 43-54. doi:10.1094/PDIS-04-11-0305. 10.1094/PDIS-04-11-0305 30731851

